# Clinical diagnosis and treatment of primary thyroid tuberculosis: a retrospective study

**DOI:** 10.1590/1516-3180.2021.0380.R1.29102021

**Published:** 2022-06-06

**Authors:** Le-Le Sun, Su Dong, Jia-Lu Xu, Jin-Xin Zhu, Jia Liu

**Affiliations:** I MD. Surgeon, Department of Thyroid Surgery, First Hospital of Jilin University, Jilin Province, China. 0000-0002-4052-6379; II MD, PhD. Anesthesiologist and Professor, Department of Anesthesia, First Hospital of Jilin University, Jilin Province, China. 0000-0001-6275-1035; III MD. Surgeon, Department of Thyroid Surgery, First Hospital of Jilin University, Jilin Province, China. 0000-0003-4295-0592; IV MD. Surgeon, Department of Thyroid Surgery, First Hospital of Jilin University, Jilin Province, China. 0000-0002-3496-0179; V MD, PhD. Surgeon and Professor, Department of Thyroid Surgery, First Hospital of Jilin University, Jilin Province, China. 0000-0003-3575-5107

**Keywords:** Thyroiditis, Tuberculosis, Diagnosis, Thyroid tuberculosis, Granulomatous inflammation, Retrospective study

## Abstract

**BACKGROUND:**

Primary thyroid tuberculosis (PTT) is an uncommon type of extrapulmonary tuberculosis, which is caused by *Mycobacterium tuberculosis*. It does not have specific clinical manifestations, and most cases are diagnosed through postoperative histopathological examination.

**OBJECTIVE:**

To evaluate the diagnostic pattern and management strategy among patients with primary thyroid tuberculosis.

**DESIGN AND SETTING:**

Retrospective study on patients with primary thyroid tuberculosis in the First Hospital of Jilin University (Changchun, China).

**METHODS:**

Between March 2015 and June 2020, nine cases of PTT were diagnosed and treated in the Department of Thyroid Surgery of the First Hospital of Jilin University. Age at diagnosis, primary symptoms, preoperative biopsy, operation method, pathological classification, acid-fast staining test, anti-TB therapy and prognosis were registered in order to explore the appropriate protocol for diagnosis and treatment of this disease.

**RESULTS:**

None of the patients was diagnosed with thyroid tuberculosis before surgery. All the patients underwent surgery. Granulomatous changes or caseous necrosis in thyroid tissue were found through postoperative histopathological evaluation. Polymerase chain reaction (PCR) results for *Mycobacterium tuberculosis* were positive in all patients. Most patients had a good prognosis after surgery and anti-tuberculosis drug therapy.

**CONCLUSION:**

PTT is a rare disease. It is important to improve the preoperative diagnosis. Preoperative diagnostic accuracy relies on increased awareness of the disease and appropriate use of preoperative diagnostic methods, such as PCR detection, fine-needle aspiration cytology, acid-fast bacillus culture, ultrasound and blood sedimentation. PCR detection of *M. tuberculosis* is recommended as the gold standard for diagnosis.

## INTRODUCTION

The World Health Organization (WHO) reported in 2009 that there were 9 million new cases of tuberculosis (TB) in the world every year. In 2011, it was estimated that there were about 8.7 million cases worldwide. It can be seen that the incidence of TB remains high and is spreading rapidly throughout the world.^1^ Thyroid tuberculosis (TT), also known as tuberculous thyroiditis, can be divided into two types: primary and secondary. Secondary thyroid tuberculosis occurs after infection with tuberculosis in any other parts of the body. Primary thyroid tuberculosis (PTT) is mostly caused by direct infection of the thyroid by *Mycobacterium tuberculosis*. Due to the lack of obvious specificity of clinical manifestations, signs and laboratory and imaging examinations, PTT can be easily misdiagnosed clinically.

## OBJECTIVE

The objective of this study was to evaluate the diagnostic pattern and management strategy among patients with primary thyroid tuberculosis.

## METHODS

In this study, the clinical data of nine patients with PTT who were diagnosed in the Department of Thyroid Surgery of the First Hospital of Jilin University between March 2015 and June 2020 were retrospectively analyzed. Age at diagnosis, primary symptoms, preoperative biopsy, operation method, pathological classification, acid-fast staining test, anti-TB therapy and prognosis were registered in order to explore the appropriate protocol for diagnosis and treatment of this disease.

### General data

Between March 2015 and June 2020, nine cases of PTT were diagnosed in the Department of Thyroid Surgery of the First Hospital of Jilin University. There were five women and four men, with a median age of 50 years (range 43-64 years) (Table 1). None of the nine patients had any history of pulmonary or extrapulmonary TB.

**Table 1.** Clinical data on nine patients with primary thyroid tuberculosis

Serial number	Sex	Age (years)	Symptoms of TB	Preoperative biopsy	Glandular lobe	Concomitant thyroid disease	Operation method	Pathological classification	Acid-fast staining test	Anti-TB therapy	Follow-up (months)	Prognosis
1	Female	58	(-)	(-)	Right	Nodular goiter	Right lobectomy + isthmus excision	Granulomatous type	(+)	Yes	70	No recurrence
2	Female	50	(-)	(-)	Left	None	Left partial resection	Granulomatous type	(+)	Yes	33	Pulmonary tuberculosis occurred
3	Male	64	(-)	(-)	Right	Nodular goiter	Right near total resection + left partial resection	Caseation type	(-)	Yes	41	No recurrence
4	Female	43	(-)	(+)	Both lobes	PTMC + Nodular goiter	Total thyroidectomy	Granulomatous type	(+)	No	52	No recurrence
5	Male	48	(-)	(-)	Right	PTMC	Right lobectomy + isthmus excision	Granulomatous type	(+)	Yes	59	No recurrence
6	Female	62	(-)	(-)	Right	None	Right partial resection	Granulomatous type	(+)	Yes	7	Sonographic features of PTT infection remain
7	Male	77	(-)	(+)	Right	Nodular goiter + lymphocytic thyroiditis	Right lobectomy + isthmus excision	Granulomatous type	(-)	Yes	22	No recurrence
8	Male	52	(-)	(-)	Right	None	Right lobectomy	Diffuse type	(+)	No	16	No recurrence
9	Female	37	(-)	(+)	Left	PTMC	Left lobectomy + isthmus excision	Caseation type	(+)	No	34	No recurrence

TB = tuberculosis; PTMC = papillary thyroid microcarcinoma; PTT = primary thyroid tuberculosis.

### Clinical manifestations

Four of the nine patients were admitted with a self-evident neck mass, and five were admitted because of thyroid nodules (nature to be determined), which had been found through outpatient physical examination. One patient presented with tenderness of the mass, but there was no obvious specific manifestation in the other eight cases. There were none of the common TB symptoms such as low fever, fatigue, night sweats, emaciation or loss of appetite (Table 1).

### Auxiliary examinations

After admission, all patients underwent routine examinations such as thyroid color Doppler ultrasonography, thyroid function tests and computed tomography (CT) scan of the lungs. Thyroid nodules were examined by means of fine-needle aspiration in some patients (3/9).

**Figure 1.**
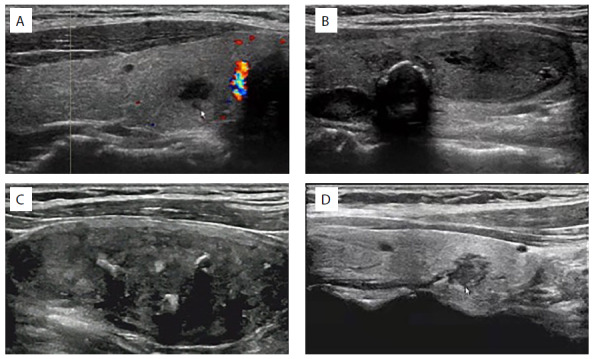
Characteristic ultrasonographic changes of primary thyroid tuberculosis. Ultrasound indicated that the thyroid mass had unclear boundaries and no obvious blood flow signal inside the mass (A); or was surrounded by a strong echo (B); or by a mostly mixed echo (C); or by a scattered strong echo in some parts (D).

Thyroid color Doppler ultrasonography showed that five patients were initially suspected of having thyroid malignancy, among whom two cases showed fine-dot calcification inside the thyroid nodule, and one case showed arc calcification at the edge of the nodule (Figure 1). The other four patients tended to be benign.

Three patients underwent fine-needle puncture cytological examination, which indicated the presence of atypical epithelial cells. The possibility of thyroid papillary carcinoma was not ruled out. No mutation of *BRAF* gene exon 15 point (V600E) was found in any of the genetic tests. The remaining six patients refused to undergo puncture examination for personal reasons, and required surgical treatment.

Serum tri-iodothyronine, thyroxine, free tri-iodothyronine, free thyroxine and thyroid-stimulating hormone levels were normal in all patients. Lung CT examination showed no obvious abnormality.

For all nine patients, intraoperative rapid frozen section examination, postoperative pathological examination of paraffin sections, acid-fast staining and polymerase chain reaction (PCR) detection of *M. tuberculosis* genes were performed.

### Treatment

None of the nine patients was diagnosed with TT before surgery and all the patients underwent surgery. Five patients were suspected of having malignancy preoperatively and underwent unilateral/bilateral lobectomy and isthmus resection (four and one cases, respectively).

In patients who underwent total thyroidectomy, rapid intraoperative pathological evaluation indicated the presence of bilateral papillary thyroid carcinoma with multiple lesions, 0.1–1.1 cm in diameter, with granulomatous inflammation (atypical epithelial cells were observed in preoperative puncture examination). Two patients underwent unilateral partial thyroidectomy after rapid intraoperative pathological evaluation indicated the presence of granulomatous thyroiditis.

Another two patients had large masses. A hypothesis of nodular goiter was considered preoperatively, and granulomatous inflammation accompanied by nodular goiter was indicated through rapid intraoperative pathological evaluation. Thus, right lobectomy resection and right near total resection + left partial resection was performed, respectively (Table 1).

### Ethics statement

These studies involving human participants were reviewed and approved by the ethics committee of the First Hospital of Jilin University (2021-087). The patients/participants provided their written informed consent to participate in this study. Written informed consent was obtained from the individual(s) for the publication of any potentially identifiable images or data included in this article.

## RESULTS

**Figure 2.**
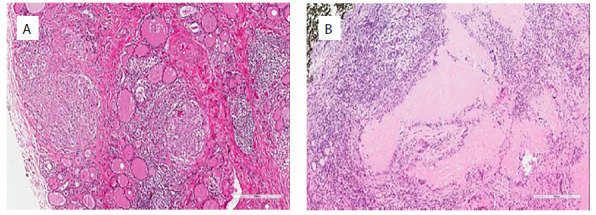
Pathological changes characteristic of primary thyroid tuberculosis. A: Diffuse multifocal irregular granuloma formation was observed in the thyroid gland. (HE 10×). B: Granuloma formation in the thyroid with caseous necrosis in the center. (HE 10×).

Presence of granulomatous changes or caseous necrosis in thyroid tissue were confirmed through postoperative histopathological evaluations on all nine patients (Figure 2). Among them, seven cases were positive for acid-fast staining and two were negative. The PCR results for *M. tuberculosis* genes were positive in all patients.

All the patients had good wound healing and were discharged after recovery. One patient who underwent total thyroidectomy and had no TB lesions in other parts of the body was only followed up closely after the operation, but the other patients were given systemic anti-TB therapy after the operation. All nine patients were followed up for 7-70 months. The disease was found to be not completely controlled in one patient, and there was recurrence of pulmonary TB in another patient. However, no recurrence was seen in the remaining seven patients (Table 1).

## DISCUSSION

PTT is a fairly rare disease and the incidence of all forms of thyroid tuberculosis (TT) is only 0.4%-0.76% in China.^2^ Here, we reported nine cases of PTT that we saw within a five-year period in our hospital. PTT is caused by *M. tuberculosis* infection and occurs when the systemic immune function is weakened.^3^

It has been reported that human immunodeficiency virus (HIV) infection or extrapulmonary TB can greatly increase the incidence of PTT, to 45%-75%.^4,5^ However, in the nine cases assessed here, there was no HIV infection or extrapulmonary TB, according to the preoperative examinations.

Misdiagnosis and missed diagnosis are common among PTT cases before surgery, and high clinical standards and experience are needed for making the diagnosis. Among our cases, the male-to-female ratio was 4:5 and the median age was 50 years old. These characteristics are not specific and are similar to what is seen regarding the incidence of thyroid disease. PTT is most frequent in the lower right lobe of the thyroid^6,7^ and the cases of our study were consistent with this (6/9).

Tuberculous nodules are often accompanied by thyroid adenoma, diffuse goiter, acute abscess and cervical lymph node lesions, and are sometimes manifested as sudden enlargement of the original thyroid nodules.^8^ Some cases are accompanied by neck compression symptoms.^9^ In a few cases, thyroid damage has been found to be caused by *M. tuberculosis* or even by thyrotoxicosis or mucoedema.^10^ Most researchers believe that the granulomatous type is the most common type in clinical practice, while a few researchers believe that the caseous type is the most common. Among the nine patients with PTT in our study, six were considered to be the granulomatous type; two, the caseous type; and one, the diffuse type.

Many studies have emphasized the importance of ultrasound in making the diagnosis of PTT. Chan et al.^11^ described the ultrasound features of a mixed cystic and solid hypoechoic mass in the right lobe. Kang et al.^12^ performed an ultrasound examination on one patient that showed enlargement of the right thyroid gland, with nodules that were mainly anechoic, with some internal echoes and irregular margins. In another patient, the left lobe showed a large, heterogeneous, mostly anechoic lesion with irregular vascular walls and a small amount of internal echo. Yang et al.^13^ carried out dynamic ultrasound monitoring on a PTT patient, and the initial ultrasound examination showed an uneven fluid-filled nodule with internal bleeding.

In the present study, all of the nine cases underwent thyroid color doppler ultrasonography, from which five patients were suspected of having thyroid malignancy and four patients tended to be benign. A single ultrasound examination was not specific for PTT in our clinical practice. Yang et al.^13^ reported that repeated ultrasound examination during disease progression showed gradual changes when the nodule was solid and hyperechoic with blurred edges.

Enhanced computed tomography (CT) scanning also plays an important role in making the diagnosis of PTT. CT may detect signs of thyroid cartilage plate destruction and laryngeal cavity narrowing and is helpful for locating caseous necrosis. Laitman et al.^14^ reported that on enhanced CT, central necrotic foci and marginal enhancement were seen in thyroid tuberculous nodules.

Some research has also described the magnetic resonance imaging (MRI) features of TT.^15^ However, we did not perform either enhanced CT or MRI on the thyroid because of their high price and narrow scope of application. Additionally, chest X-rays and abdominal ultrasonography can be used to detect TB in other parts of the body. In our study, all patients underwent CT scans of the lungs to rule out pulmonary tuberculosis.

At present, fine-needle aspiration cytology (FNAC), acid-fast bacillus culture and PCR detection of *M. tuberculosis* are the most important means for preoperative diagnosis. On the other hand, acid-fast staining in PTT patients has been found to present a high false-negative rate.^16^ Therefore, PCR detection of *M. tuberculosis* is now recommended as the gold standard for diagnosis. However, among our cases, six patients out of nine refused to undergo FNAC before surgery because they were prone to excessive anxiety or worried about malignant changes and insisted on undergoing the operation directly.

For patients without a definite preoperative diagnosis, rapid intraoperative frozen section examination should be performed, so as to facilitate selection of surgical methods and avoid excessive surgery. In cases of PTT that undergo total thyroidectomy, the examination must be performed to rule out the presence of other focal infections throughout the body. If no other lesions are found, close follow-up is recommended, and additional anti-TB therapy is not required.^17^ However, if other lesions are present, at least six months of anti-TB therapy should be given, even if total thyroidectomy is performed. If TB is found in patients who have undergone subtotal or near-total thyroidectomy and thyroid lobectomy, anti-TB therapy should be given for at least six months, regardless of the presence of any additional lesions. Among our patients, only one patient underwent total thyroidectomy without anti-TB therapy, and the remaining eight received postoperative anti-TB drug therapy. The follow-up showed no recurrence in any of the patients.

For patients with complications such as abscesses or sinus passages due to TB, drainage or complete thyroidectomy with medication is recommended. This medication should be continued for at least an average of four months after the last operation. The appropriate follow-up for patients with PTT includes ultrasonography every three months for the first six months, followed by ultrasonography every six months for two years, and then annually thereafter.^17^ Among our patients, only one patient underwent total thyroidectomy without anti-TB therapy, and the remaining eight received postoperative anti-TB drug therapy. The follow-up showed no recurrence in any of the patients within follow-up periods ranging from 7 to 70 months.

## CONCLUSION

Primary thyroid tuberculosis is a rare thyroid disease and it is easy to misunderstand the diagnosis, thus resulting in delayed treatment. Here, we presented our diagnosis and treatment experiences relating to nine cases that were confirmed as primary thyroid tuberculosis through postoperative histopathological evaluations. We believe that with further accumulation of cases and experience in the future, our understanding of PTT will become enhanced, and the diagnosis and treatment of this disease will be improved.
